# Length and Dimensional Measurements at NIST

**DOI:** 10.6028/jres.106.002

**Published:** 2001-02-01

**Authors:** Dennis A. Swyt

**Affiliations:** National Institute of Standards and Technology, Gaithersburg, MD 20899-8201

**Keywords:** atomic-force, dimensional, interferometry, length, measurements, microscopes, optical, scanning-electron, scanning-tunneling, traceability

## Abstract

This paper discusses the past, present, and future of length and dimensional measurements at NIST. It covers the evolution of the SI unit of length through its three definitions and the evolution of NBS-NIST dimensional measurement from early linescales and gage blocks to a future of atom-based dimensional standards. Current capabilities include dimensional measurements over a range of fourteen orders of magnitude. Uncertainties of measurements on different types of material artifacts range down to 7×10^−8^ m at 1 m and 8 picometers (pm) at 300 pm. Current work deals with a broad range of areas of dimensional metrology. These include: large-scale coordinate systems; complex form; microform; surface finish; two-dimensional grids; optical, scanning-electron, atomic-force, and scanning-tunneling microscopies; atomic-scale displacement; and atom-based artifacts.

## 1. Introduction

One of the most venerable, commonly encountered, scientifically fundamental, and economically important units of measure is length. It is one of the fundamental measurement quantities in physics, commerce, and everyday life. The international standard of length is the meter, one of the seven base units of the modern International System of Units (SI) and one of the two original units of the international system of standards upon which the SI is based. Both the meter as the unit of length and dimensional measurements based on the meter have undergone substantial changes over the lifetime of the National Bureau of Standards and its successor, the National Institute of Standards and Technology.

### 1.1 The Evolution of the Meter Since 1901

Three different definitions of the international standard of length have been in effect during the lifetime of NBS-NIST. At the time of the founding of the National Bureau of Standards in 1901, the international standard of length was the International Prototype Meter. The meter was defined at that time as the distance between two lines ruled on a platinum-iridium bar carefully preserved in a special vault at the International Bureau of Weights and Measures (BIPM) near Paris [1]. With its founding, NBS became the keeper of a duplicate of this bar, Meter No. 27, which then served as the U.S. national standard of length for 60 years. At the end of that period, the meter as the international standard of length underwent the first of two fundamental re-definitions.

#### 1.1.1 The Re-Definitions of the Meter

In 1960, the meter was re-defined by the General Conference on Weights and Measures (CGPM) to be 1 659 763.73 vacuum wavelengths of light resulting from the unperturbed atomic energy level transition 2*p*_10_–5*d*_5_ of the krypton isotope having a relative atomic mass of 86 [2].

In 1983, the meter was re-defined again to the one in effect today, namely: “The meter is the length of path traveled by light in vacuum during the interval of 1/299 792.458 of a second” [3]. (Among the effects of the definition is that it fixes the speed of light in vacuum to be exactly 299 792.458 meters per second). At that time, the International Committee on Weights and Measures (CIPM) gave three basic methods for the practical realization of the meter: time-of-flight, using time intervals, and interferometry, using wavelengths or frequencies. CIPM gave five recommended radiations with assigned frequencies, wavelengths, and uncertainties. Of the recommended radiations, that of the iodine-stabilized helium-neon laser is the most widely used for practical realization of the meter. It has a wavelength of *λ*_HeNe_ = 632.991 398 22 nm, with a relative standard uncertainty *u*_r_ of 2.5×10^−11^ [4].

The effect of the re-definitions and advances in measurement of the frequencies of recommended radiations was to decrease the relative uncertainty attainable in realization of the meter by five orders of magnitude

- from an estimated 2×10^−6^ (this paper’s estimate of the reproducibility with which the first transfer could be made from the prototype meter bar) [5],
- through 7×10^−8^ (the relative uncertainty for the wavelength emitted by cadmium discharge lamps, a secondary standard of length),
- through 4×10^−9^ (the relative uncertainty for the wavelength emitted by krypton-86 discharge lamps),
- to 2.5×10^−11^ (the CIPM specified uncertainty for the visible wavelength of the iodine-stabilized helium-neon laser today) [4].

#### 1.1.2 NIST Contributions to the Re-definitions of the Unit of Length

The unit of length has evolved from a definition based on a physical prototype through one based on a specific wavelength of light to one based on an electromagnetic wave propagating in free space. NIST has made substantial contributions to this evolution. These contributions include:

- Production in 1947 of isotopically pure mercury-198, measurement of its spectral linewidth and proposal of its wavelength for adoption as the international standard of length [6];
- Measurement in 1971 of the spectral linewidth and frequency of an emission line of a helium-neon laser corresponding closely to an absorption line of iodine, then a candidate for a recommended radiation for the re-definition of the meter to replace that of krypton-86, the standard for definition of the meter at the time [7];
- Measurement in 1976 of the ratio of the wavelength of an iodine-stabilized HeNe laser to that of a methane-stabilized He-Ne laser, providing a provisional extension of the frequency scale based on the cesium oscillator into the visible spectrum [8];
- Development in 1980 of a portable iodine-absorption-stabilized helium-neon laser for use in international metrology [9];
- Measurement in 1983 of the frequencies of visible-light lasers, including that of the iodine-stabilized laser, directly against that of the cesium-beam atomic clock, the primary standard of time [10].

### 1.2 The Evolution of Dimensional Metrology Since 1901

The definition of the meter—whether in terms of a prototype meter bar, a wavelength of light, or the propagation of an electromagnetic wave in an interval of time—has provided the basis for the lowest-uncertainty realization of the unit. A primary economic driver for reduced uncertainty with which the meter could be realized has been demands for reduced uncertainty in measurements made in commerce, especially by manufacturers using leading-edge technology in the production of goods. These measurements are not of the “Platonic length” of wavelengths of light propagating in free space but of the physical lengths of material objects, from aircraft wings and automobile engine parts to microelectronic devices. Measurements of dimensions of material goods are most often referenced to the SI unit of length through material artifacts calibrated as dimensional standards. NIST has played a key role for the United States as provider of the link between the Platonic length of the laboratory and the physical length of material objects through its practice of dimensional metrology.

#### 1.2.1 Two Historical Dimensional Measurements

Two mainstays of NIST dimensional metrology over the lifetime of NBS-NIST have been measurements of linescales and gage blocks.

##### 1.2.1.1 Measurement of Linescales Since 1901

The lowest uncertainty attained in dimensional measurement of a material object occurs in the calibration of linescales. The dimensional feature of interest in a linescale is the distance between parallel lines inscribed on a substrate.

By 1904, NBS was providing calibrations of linescales relative to the U.S. prototype meter bar for scales from 100 mm to 50 m in length with subdivisions down to 0.1 mm [6]. Today, NIST provides calibrations of linescales relative to first-principles realizations of the meter using displacement interferometry. These calibrations range from scales as small as 10 μm in length (with subdivisions down to 1 μm) to as long as 50 m (with subdivisions down to 0.1 mm) [11].

Changes have occurred over the century in how NBS-NIST has stated its estimate of the closeness of the value of the quantity being measured to the result of a measurement—from no statement, to that of maximum likely error, to accuracy, and now to uncertainty. As a result, it is not possible to estimate the standard uncertainty of measurement results for those reported over the period. However, a reasonable characterization is that:

- For the period from 1904–1960, the *reproducibility* of measurements against the U.S. prototype meter bar is estimated to be of the order of 0.25 μm, in relative terms, 2.5×10^−7^ at 1 m, with the legibility of the lines on the bar the major limitation [5].
- For the period from 1960–2000, the *expanded uncertainty U* (coverage factor *k* = 2) for measurements of one-meter linescales by interferometry against a wavelength of light decreased progressively from 0.25 μm in 1960 to 0.08 μm (8×10^−8^ at 1 m) today, due to improvements in measuring machine geometry, light sources, and temperature measurement and control [11].

**Fig. 1:**
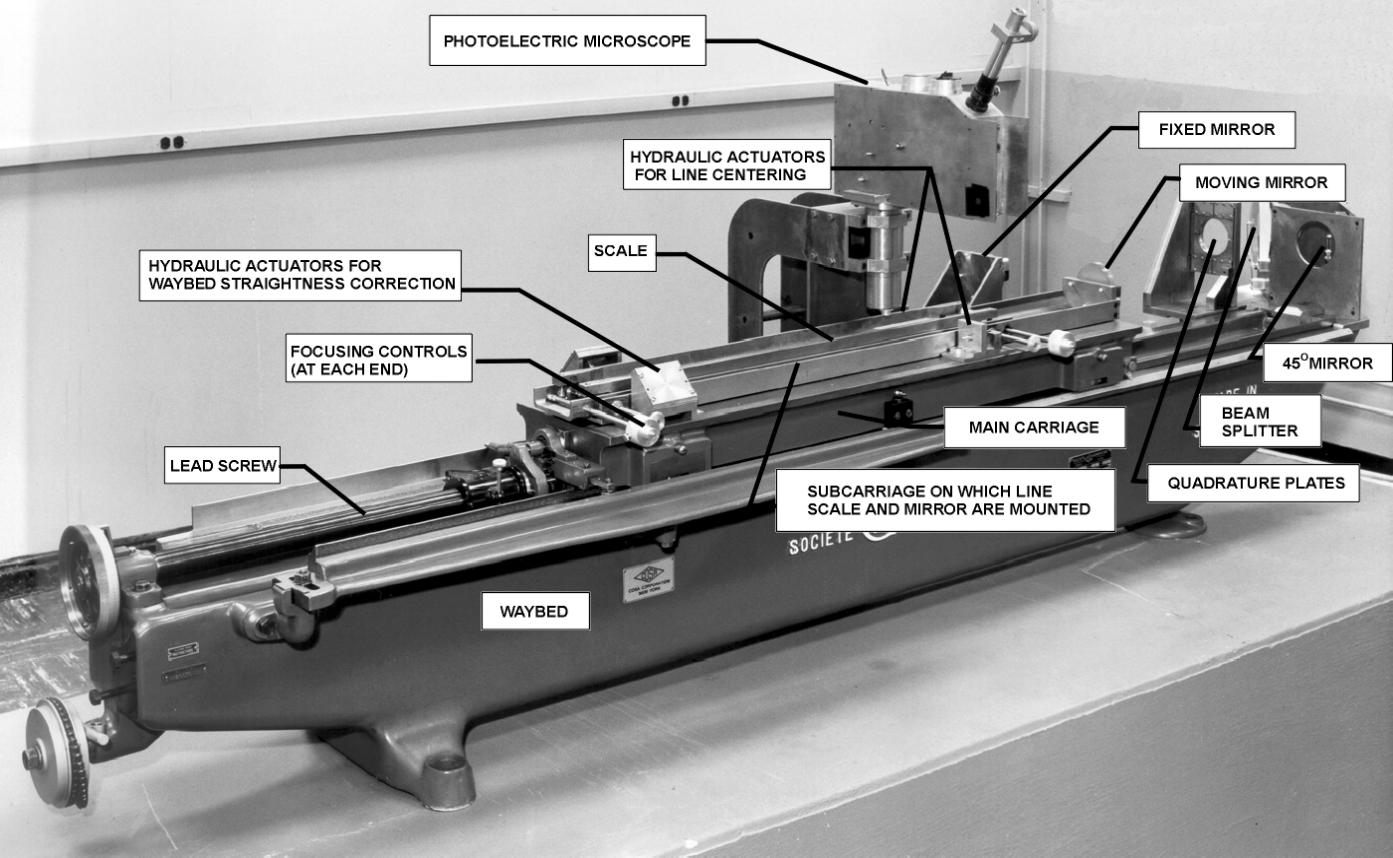
The NIST line scale interferometer system as it appeared starting in 1971 [11]. It was first introduced into service in 1965.

Figure 1 shows the NIST Line Scale Interferometer System, first introduced in 1965, as it appeared in 1971.

##### 1.2.1.2 Measurement of Precision Gage Blocks Since 1901

One of the most industrially important length-measurement standards, particularly for machine-tool-based manufacturing, is precision gage blocks. Consisting of blocks of metal, usually steel, having two opposite faces that are plane, parallel, and a specified distance apart, they are used in manufacturing as size blocks for precise mechanical work and for checking precise mechanical work.

Prior to 1917, NBS is reported to have been calibrating precision gage blocks with mechanical-contact comparators against end standards calibrated by visual-microscope comparison to linescales calibrated by visual-microscope comparison to the U.S. prototype meter bar. Based on the “error” in the process then reported, today’s estimate of the uncertainty of those earliest NBS calibrations of precision gage blocks is 0.75 μm (7.5×10^−4^ at 1 mm).

In 1922, NBS introduced its first interferometric measurements of gage blocks, reducing the estimated uncertainty by an order of magnitude to 0.075 μm (7.5×10^−5^ at 1 mm). In 1935, NBS reportedly gained another factor of three improvement to an estimated uncertainty of 0.025 μm (2.5×10^−5^ at 1 mm). With other improvements, especially improvement of the geometry and material-stability of the blocks in 1960 [6], the limiting expanded uncertainty (coverage factor *k* = 2) for short blocks today is 0.008 μm (8×10^−6^ at 1 mm) [12], an improvement of two orders of magnitude over the lifetime of NBS-NIST.

#### 1.2.2 Some NIST Contributions to Dimensional Metrology Since 1901

NBS has made fundamental contributions to the evolution of dimensional measurements over the period since the founding of NBS to the era of current work, which reaches back to the beginning of the last decade of the twentieth century. These fundamental contributions include:

- Introduction in 1922 of interferometric measurements of precision gage blocks [13]
- Development in 1961 of high-stability precision gage blocks [6]
- Creation in 1968 of the first scanned probe topography measuring instrument, a field-emission device that was the precursor of the scanning tunneling microscope and that was cited in the Nobel Prize award for that device [14]
- Development in 1976 of the technique for the low-uncertainty optical-microscope measurement of microelectronic photomask linewidths [15]
- Development in 1977 of the technique of computer-based real-time correction of systematic errors in positioning of coordinate measuring machines [16]
- Development in 1981 of the technique for laser-interferometer-based scanning-electron-microscope measurement of microelectronic photomask linewidths [17]

### 1.3 The Industrial Driver for Lower Uncertainties in Standards: Tightening Tolerances

The need for reduced uncertainty in the “primary standard” aspect of length, that is, in its definition and realization, and in the “secondary standard” aspect, that is, in its transfer and dissemination through dimensional metrology, is linked strongly to tightening tolerances in industrial manufacturing.

#### 1.3.1 NIST Uncertainty Relative to Industry Tolerances

The basic logic is that measurements made by NBS-NIST as the national metrology institute responsible for realization and dissemination of the SI unit of length need to be at levels of uncertainty that are small fractions of the tightest tolerances achieved in manufacturer’s use of leading-edge technology. NBS length metrologists’ explicitly used this line of reasoning within two decades of NBS’ founding [13]. It is still valid today.

In order to assess with confidence the conformance of parts to tolerances, the uncertainty associated with the gages employed was required to be some fraction of the tolerance on the dimensions of the part being measured. In other words, the uncertainty associated with measurements made with the gage was required to be equal to the value of the tolerance divided by some factor. In a 1918 treatise on industrial measurement and inspection, the gage uncertainty was required to be less than the part tolerance by a factor of four (or five, depending upon round-off to the nearest half-digit) [18]. By the same reasoning, the uncertainty of the process of calibration of the gage was required to be a second factor of four smaller than the desired gage uncertainty.

According to a 1922 NBS paper on interferometric measurement of gage blocks [13], NBS’ calibration of the testing laboratory’s standards was, in turn, required to be a third factor smaller than that of the gage uncertainty. As a result of these three successive reductions by factors of four or five (less round-off at various levels), the uncertainty required of NBS calibrations at that time was deemed to be of the order of 1/100 of the more demanding part tolerances of the day.

Now a common machining tolerance of the time was reportedly ±50 μm [18] and the uncertainty of NBS calibrations of gage blocks prior to 1917 was 0.5 μm to 1.0 μm [13]. Thus the lower end of the NBS uncertainty was smaller by the requisite factor of 100 than the commonly called-for tolerance (presumably a high-accuracy tolerance for an earlier decade). By 1917, however, the tolerance of a high-accuracy part was ±6.25 μm [18], and, tolerances of ±2.5 μm were being sought [13]. In order to provide calibrations a factor of 100 better than that latter tolerance, NBS advanced its measurement capabilities to provide calibrations of gage blocks with an uncertainty of the required ±0.025 μm [13].

#### 1.3.2 The Trend of Tightening Tolerances

The trend of tightening tolerances and the consequent need for lower uncertainties at NBS-NIST as first suggested in 1922 [13] have continued unabated throughout the lifetime of NBS-NIST. According to an 1980 academic analysis of industrial trends in ultra-precision machining over the central decades of the twentieth century, achievable machining tolerances for particular classes of processes has decreased at a rate of approximately an order of magnitude every twenty years [19]. By this account, the tolerances achievable by what is described as normal precision machining have decreased from the order of 10 μm in the period 1920 to 1940 to less than 1 μm in the period 1980 to today. The analysis also indicated an evolution of a parallel, ultra-precision machining regime—which includes atomic-, molecular-, and ion-beam milling and semiconductor-lithography processes—that has tolerances an order of magnitude smaller than those of the normal precision regime. In this ultra-precision regime, attainable tolerances have decreased from the order of 1 μm in the period 1920 to 1940 to the order of 1nm to 10 nm today.

## 2. Dimensional Metrology at NIST Today

Today, the NIST division responsible for the realization and dissemination of the SI unit of length serves a range of industries, from aircraft and automotive to computers and microelectronics. It provides fourteen major types of length measurement services to approximately 120 different fee-paying institutional customers per year. Each measurement service begins with a first-principles realization of the SI unit of length via frequency-stabilized lasers and displacement interferometry. The measurement technologies employed include laser-ranging devices, theodolites, large-scale coordinate measuring machines (CMMs), optical- and ultraviolet-light microscopes, scanning electron microscopes (SEMs), atomic force microscopes (AFMs), and scanning tunneling microscopes (STMs).

### 2.1 The State of NIST Dimensional Measurement Services

Table 1 describes a number of the types of length measurements provided by NIST today. Shown in the table for each type are: range; expanded uncertainty; relative expanded uncertainties at respective ends of the range; and an assessment of where the uncertainty stands relative to the best provided by other national metrology institutes (NMIs).

Representing the largest dimensions that NIST calibrates are surveyor’s measuring tapes, one type of linescale. The 50 m length of such measuring tapes can be calibrated to an expanded uncertainty (coverage factor *k* = 2) of 500 μm or, fractionally, 1×10^−5^ at 50 m. According to a benchmarking of NIST measurement services against those of eleven other NMIs, including all of the major industrialized countries, these uncertainties tie NIST with one other NMI for providing the lowest uncertainty [20].

Representing the lowest relative uncertainty (*U/L*) of dimensional measurements provided in a NIST calibration is that of the length of a 1 m linescale. In this case, the relative expanded uncertainty (coverage factor *k* = 2) is 7×10^−8^ at 1 m [11]. According to the NIST benchmarking study cited, this is also the lowest uncertainty of a dimensional measurement of a material artifact provided by any of the world’s NMIs [20].

Representing the lowest uncertainty of linescale measurements is that on the 1 μm subdivision of a scale of 10 μm in overall length. The attainable expanded uncertainty (coverage factor *k* = 2) for these short linescales is 1 nm [11]. According to the NIST benchmarking study, this is also the lowest absolute uncertainty of a linescale measurement provided by any of the world’s NMIs [20].

Representing the lowest relative uncertainty of an end standard is that of the 1 m step on a CMM step gage [12]. According to the NIST benchmarking study, with its relative expanded uncertainty (coverage factor *k* = 2) of 7×10^−7^, NIST is tied with one other NMI in providing this level of uncertainty [20].

Representing the state-of-the-art of precision gage block calibration is the expanded uncertainty of 10 nm to 30 nm on gage blocks of 10 mm to 1000 mm in length [12]. According to the NIST benchmarking study, the NIST uncertainty is that attained by the group of the leading NMIs of the world [20].

Representing the lowest uncertainty of end-standard-type measurements in the microscopic regime is that of sub-micrometer and micrometer linewidths of the NIST photomask linewidth standards, with an expanded uncertainty of 36 nm over the range of lines from 0.5 μm to 30 μm width [23]. According to the NIST benchmarking study, NIST is the first provider of such standards and provides the lowest uncertainty [20].

Finally, representing the lowest reported uncertainty ever attained in an SI-traceable dimensional measurement of an individual material feature is that of the step height of fabricated single-atom steps of silicon (111). The expanded uncertainty (coverage factor *k* = 2) of measurement of the 304 picometer (pm) step height is 8 pm [24, 25].

### 2.2 Research and Development in Dimensional Metrology at NIST Today

Given the trend to tightening tolerances in precision machining and the goal of a factor of 100 for NIST to surpass the tightest tolerances in the manufacturing it supports, NIST would be expected to provide measurements with uncertainties of the order of tens of nanometers to support what has been called the “normal precision machining” regime and of the order of tens of picometers to support the “ultra-precision” regime. For one particular standard for each regime, NIST can be viewed as meeting those projections. For the normal machining regime, NIST provides calibrations of precision gage blocks with a state-of-the-art expanded uncertainty (coverage factor *k* = 2) of 10 nm. In the “ultra-precision machining” regime, NIST can perform measurements of single-atom steps in silicon with an expanded uncertainty of 8 pm. At the same time, NIST is carrying out extensive research and development to address anticipated U.S. industry needs for new types of dimensional measurements and reduced uncertainties.

#### 2.2.1 The First-Principles Method of NIST Dimensional Measurements

Today, possibly more so than at any time in its history, NIST is called upon to meet extraordinary demands of U.S. manufacturing industries in their use of leading-edge technologies with state-of-the-art dimensional tolerances. These extraordinary demands include:

1. uncertainties for dimensional measurements on production devices that are beyond the world state-of-the-art in measurement capability [26]; and
2. traceability to a measurement by an NMI of a “primary standard” of the particular dimensional feature of their discrete-part product, that is, what is now being called measurement-task-specific traceability [27]; and, in some cases.
3. both state-of-the-art uncertainty and NMI traceability in the same measurement.

Demands from industry for NIST to develop low-uncertainty, task-specific, “primary-standard” measurements often arise when there is an unresolved discrepancy between different, highly reproducible results of measurements made respectively by producers of and customers for economically important products with state-of-the-art dimensional tolerances. The circumstances of such an unresolved discrepancy in measurement results are frequently as follows:

- In order to achieve a critical function of a business-critical product, a company (in this scenario, one in an economically important industry) designs a part to a tight dimensional tolerance.
- A manufacturer produces the critically dimensioned part.
- In order to achieve the tight tolerance, the manufacturer uses a manufacturing process that produces parts to high precision with high reproducibility.
- To assure conformity of the part to the customer-specified tolerance, the manufacturer makes measurements of the part’s critical dimension with a high-resolution measuring instrument, often the best commercially available.
- The customer also makes measurements of the part’s dimension, with a comparable or identical measuring instrument.
- The results of the manufacturer’s measurements and of the customer’s measurements are of high precision and high reproducibility.
- The results of the manufacturer’s measurements indicate that the part dimension is within specified tolerance.
- In contrast, the results of the customer’s measurements indicate that the part dimension is out of tolerance.
- To the manufacturer, the part conforms to specification and is acceptable.
- To the customer, the part fails to conform to specification and is unacceptable.
- The discrepancy in the measurement results cannot be accounted for by the manufacturer and the customer.

In sum, the situation is a market-transaction disagreement between sets of results of high-precision, high-reproducibility measurements made with state-of-the-art measuring instruments on parts with state-of the-art-tolerances.

For NIST to contribute to the resolution of such disagreements requires that NIST fundamentally advance the state of the art of measurement science and technology. Prototypical results of NIST to resolve such discrepancies are its photomask linewidth Standard Reference Materials (SRMs) and its gear-form calibration services.

Over the last two decades, NIST has developed a family of photomask linewidth standards covering a range of linewidths measured by optical [15] or scanning electron microscopes [28] from 30 μm down to 0.25 μm. More recently, NIST has developed calibration services for the dimensions and geometrical forms of involute gears that are critical parts of transmission power trains of aircraft, heavy equipment, and automobiles [29].

The prototypical solution to the problem of systematic differences in measurement results of dimensions produced by different dimensional measuring instruments is calibration of the instruments against the same reference standard. The requirement for the standard is that its measurement uncertainty be much smaller than the discrepancies in question.

Historically, the uncertainty associated with gages or inspection machines is required to be factors of 4, 5, or even 10 times smaller than tolerances. In turn, the uncertainty associated with industry reference standards is required to be factors of 4 to 10 times smaller than gage or inspection machine uncertainty. Finally, the uncertainties of NIST dimensional standards are expected to be factors 4 to 10 times smaller yet again [30]. Thus the uncertainties of reference measurements or calibrated standards sought from NIST can be factors of 64 to even 1000 times smaller than state-of-the-art tolerances.

The ability of NIST to provide reference measurements at such levels of uncertainty requires developments beyond the current state of the art in each of three areas:

- the physical artifact to be calibrated;
- the measuring machine to do the calibration
- the theoretical model of the systematic errors in measurement results arising from the interaction of the artifact and the measuring machine in the calibration process.

In addition, the three developments need be tied together in a measurement procedure that includes innovative measurement algorithms and methods.

##### 2.2.1.1 The Artifact

The innovative physical artifact that NIST needs to develop in order to provide reference measurements to deal with the scenario described above is one that mimics the product features for which industry is experiencing the discrepant measurement results. This artifact is required to be of a material and a form and have features and dimensions similar to the dimensioned part that is at issue in the industry. Because the artifact is used in two sets of measurements, variations in its dimensioned features contribute to a user measurement uncertainty twice: once in its calibration by NIST and once again in its use for calibration of a user’s instrument. As a result, the variations in the features are required to be substantially smaller than the measurement uncertainty required of NIST. Ideally, variations in features would be so small as to contribute insignificantly to the measurement uncertainty NIST delivers. By the same token, these variations should be substantially smaller than the variations of the manufactured part in question. Since the product is the result of state-of-the-art manufacturing processes, the artifact often needs to be of a degree of geometric perfection beyond the current state of the art.

The historical prototype of the idealized-geometry physical artifact as the basis for low-uncertainty calibrations by NBS-NIST is the industrial precision gage block. Gage blocks were invented and developed by others between 1910 and 1920 and substantially improved by a NIST-industry collaboration in the 1950s [13, 6]. Modern counterparts to gage blocks are the NIST photomask linewidth standard [23], the NIST sinusoidal surface-roughness standard [31,32], and the NIST microelectronic overlay standard [33]. Each of these artifact standards, developed during the last two decades, required advancing the state-of-the-art of manufacturing processes for its production.

##### 2.2.1.2 The Measuring Machine

For a NIST measurement process to be capable of resolving the discrepancies encountered by industry in its measurement processes, the NIST measurements need to be highly reproducible and free of the systematic errors implicit in industry’s reproducible but discrepant results.

At the heart of each of NIST’s industry-problem-solving measurement processes is an innovative, specialized, first-principles measuring machine. The innovative aspect of the machine is its ability to make measurements with uncertainty previously unattainable for that specific task. The specialized aspect of the machine is its ability to make task-specific measurements, such as that of photomask linewidth, gear involute, or machined-part cylindricity, over a particular range of feature dimension. The first-principles aspect of the machine is its direct realization of the definition of the SI unit of length in the task-specific dimensional measurement it is designed to perform. Practical realization of the definition of the meter (Sec. 1.1.1) in a dimensional measurement most commonly implies that one must be able to do three things:

- generate a line in space
- define the end points of that line
- divide the interval of space between the end points of that line into appropriate subintervals.

To carry out these functions, a measuring machine needs to have certain essential elements [34].

###### Frame

The first element is the means for the physical definition of a line in space. Geometrically, a line is defined by a direction in space relative to a coordinate system having axes and an origin. The frame is the set of physical elements that define physical points, lines, and planes to embody, to the degree of perfection required, the ideal geometry of that reference coordinate system. In general, axes are generated by a variety of mechanical devices that constrain motion in all but one direction such as v-groove ways, while an origin is generated by a well-defined mechanical stop.

###### Motion Generator

The second element is a set of physical structures, such as a moving stage or an image scanner, to generate reproducible relative motion between the object of measurement and the coordinate frame. This motion may be actual or virtual. Actual motion is by means of a physical carriage that translates the object relative to a stationary frame or translates the frame relative to the stationary object. Virtual motion may be, for example, by means of translation of an image of the object relative to the coordinate frame.

###### Probe

The third element is a probe, that is a sensor system that simultaneously detects a boundary, such as an edge or surface, of the object to be measured and locates that detected feature relative to the coordinate system. The physical principles underlying probes on NIST first-principles dimensional measuring machines include: the mechanical-contact of a gage-block comparator, the reflected visible light of a linescale optical microscope, the scattered electrons of a metrology SEM and the quantum-mechanical tunneled electrons of an STM. In each case, the probe, in effect, defines the end points of the line segment implied in the “length of path” portion of the definition of the unit of length.

###### Interval and Subintervals

The last element is the means for determining an interval or subintervals of distance in terms of the definition of the meter. The means is to use the known wavelength of a reference laser and laser displacement interferometry. The reference laser is typically a commercial, frequency-stabilized, HeNe laser calibrated against an iodine-frequency-stabilized HeNe laser, one of the recommended radiations for the practical realization of the meter. Since the definition of the meter fixes the speed of light in vacuum to be exactly 299 792 458 meters per second, and the relation of the wavelength of an electromagnetic radiation to its frequency is *λ* = *c/v*, by measuring the frequency of a laser with a given relative uncertainty, one immediately knows its wavelength with the same relative uncertainty.

Table 2 describes the type of probe, frame, scales and length reference for each of six different dimensional measuring machines at NIST, each of which embodies the elements for the realization of the meter as the SI unit of length.

**Fig. 2:**
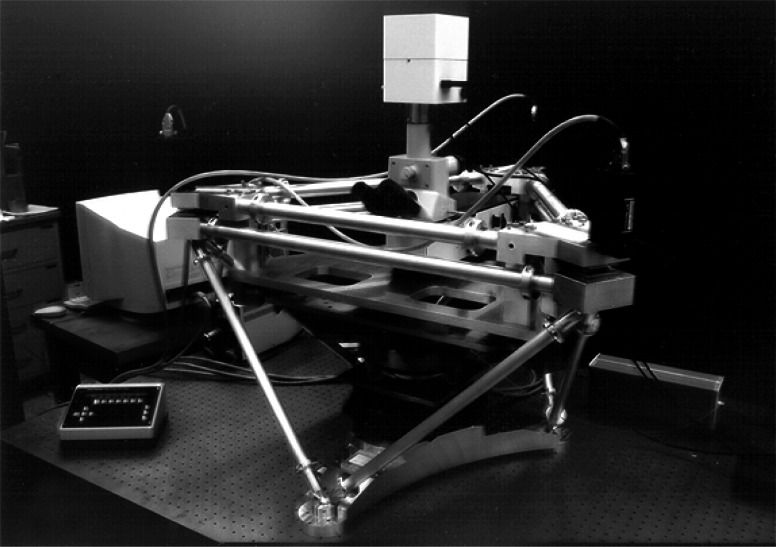
The NIST optical overlay microscope, utilizing an innovative Stewart-platform structure and digital-array image processing [33]

- The NIST coordinate measuring machine °CMM) for measuring industrial gages uses a mechanical-contact probe, an *x-y* slideways stage and *z*-axis ram, and helium-neon laser displacement interferometers for each axis [12].
- The NIST gage block interferometer for calibration of precision gage blocks is a single z-axis Michelson interferometer with a bridge over a fixed platen [22].
- The NIST overlay microscope, shown in Fig. 2, is a visible-light-microscope system with an *x-y* stage with moveable *z*-axis, with helium-neon laser displacement interferometers on each axis for calibration of microelectronic overlay error standards [33].
- The NIST metrology SEMs are scanning electron microscopes with single-axis stage-interferometers system for calibrating 250 nm photomask linewidths [28] and high-accelerating-voltage SEM magnification standards [35].
- The NIST Calibrated Atomic Force Microscope (C-AFM) has laser displacement interferometers on each of its *x* and *y* axes and a laser-interferometer-calibrated capacitance gauge on its *z* axis, for calibration of nanometer-scale step-height, pitch, and roughness standards [24].
- The NIST Molecular Measuring Machine (M3) is a scanning-tunneling-microscope-based system being developed for nanometer-uncertainty measurements over a 50 mm by 50 mm area [36].

##### 2.2.1.3 The Theoretical Model

In addition to artifacts and measuring machines, NIST measurements to address industry’s most fundamental measurement problems require theoretical models that advance the state-of-the-art. Such models are most often needed to scientifically understand the interaction between the artifact and measuring machine in order to eliminate systematic errors in measurement results due to that interaction. The source of the systematic error is in the physics that governs the interactions of the probe with the material boundary of the feature to be located. Probe-boundary interactions contribute to errors in length measurements depending upon the type of length being measured.

In dimensional metrology, there are four fundamental types of “lengths” [34]. *Extension* is the scalar quantity that describes the length of path in space between the locations of two opposite-facing boundaries of one object. *Displacement* is the vector quantity that describes the length of path in space between the locations of a single object at two different times. *Position* is the vector quantity that describes the length of path in space between the center of one object and an origin or coordinates equivalent to a second, reference object. *Distance* is the scalar quantity that describes the length of path in space between the centers of two objects. Each of the latter three types of length measurements is, in effect, the distance between either point-like or centroid-type features.

Extension-type measurements are most susceptible to probing errors and require the greatest degree of theoretical understanding in order for them to be carried out to low uncertainty. For displacement, position, and distance type measurements, probing errors at successive boundaries tend to be subtractive, canceling each other out. For extension-type measurements, probing errors at successive boundaries tend to be additive, reenforcing each other and creating systematic errors in resulting measurements. Automobile-engine cylinder bores, communication optical-fiber diameters, and microelectronic photomask linewidths are extension-type measurements. For low-uncertainty measurement results to be achieved in such measurements, the systematic errors inherent to them must be identified, theoretically modeled, and removed.

Theoretical models developed by NIST to make low-uncertainty reference measurements have accounted for systematic errors in

- the interaction of the mechanical response of a class of commercial CMM stylus probe used in CMM measurements of calibration ball bars [37]
- interaction of visible light reflected from chrome-on-glass lines in the optical-microscope measurement of the optical photomask linewidth [15]
- the emission of secondary electrons scattered from metal-on-silicon lines in the SEM measurement of the electron-beam and x-ray mask linewidths [28].

In addition, theoretical modeling has been developed to deal with systematic-error effects by

- group-theory-based estimation of finite dimensions and geometry of probes in scanned-probe-microscope measurements of surface morphology [38]
- Monte-Carlo simulation of the uncertainty of CMM measurements [39]
- a Bayesian-statistics method for calculation of measurement uncertainty using prior information [40]
- measurement uncertainty in the presence of uncorrected bias [41]

##### 2.2.1.4 The Measurement Algorithm

Finally, state-of-the-art measurements require specialized measurement techniques, including measurement algorithms and procedures, to define the task-specific measurement quantity, or measurand, of the measurement process. Current work has recently developed

- a methodology for calibrating high-resolution two-dimensional grids [42]
- a technique for measuring interferometric phase shifts of gage blocks [22]
- a technique for low-uncertainty calibration of cylinder diameters [43]
- algorithms for calculating single-atom step heights [44]
- a method to determine linewidth based on counting the atom spacings across a line [45].

#### 2.2.2 Needs of Some Key Industries in Dimensional Metrology

At present, the aircraft, automobile, computer, and microelectronic industries are representative of the industries that NIST work in dimensional metrology impacts.

##### 2.2.2.1 Aircraft Industry

With the decline in defense spending world-wide and increasing global competition in the aircraft-aerospace industry, U.S. aerospace firms are looking to export markets for survival and growth. As a result, such firms see a need to adopt international specifications in order to achieve higher levels of demonstrable quality and performance standards and as a basis for sales and procurements and [46]. Tolerances are tightening in components and assemblies of the airframe and mechanical systems, with reduction in dimensional variability aimed to attain fits in fuselage assemblies without the historical practice of using shims [47]. These tighter tolerances include, for example, specifications of fastener-hole locations on a 35 m wing to ±750 μm (2×10^−5^) [48]. For comparison, Table 1 shows the expanded uncertainty (*k* = 2) for a NIST calibration of survey tapes at that distance, which is itself only 1×10^−5^.

##### 2.2.2.2 Automotive Industry

With rapid globalization of the world’s auto industry, international standards are altering the way business is conducted throughout the world [46]. With higher customer expectations for fit and function, automobile engines and drive trains now have the same micrometer dimensional tolerances associated with the finest mechanical-movement timepieces. Some representative tight tolerances in the automobile industry today include: ±250 μm assembly tolerances on 5 m luxury-class automobile bodies; ±7.5 μm size tolerances on 96.5 mm engine piston bores; and ±0.25 μm gap tolerances on gasoline fuel-injectors [48]. To support such tolerances, the automobile industry and the measuring instrument manufacturing industry are seeking lower-uncertainty standards in each of those areas.

##### 2.2.2.3 Computer Industry

Hard-disk-drive technology, the pacesetter for the computer data storage industry, like electronics, is driven by competition to follow Moore’s law in shrinking dimensions and tightening tolerances [49]. Hard disk drives are exhibiting a compound annual growth rate of 60 % for areal information density, corresponding to decreases in dimensions and tolerances of 30 % per year [50]. Today, hard disk drives involve design and fabrication of topographic structures of a few micrometers, lateral dimensions less than a micrometer, and film thicknesses of a few nanometers. The trend is for reduction of critical dimensions and tolerances on magnetic heads by a factor of 5 between 1997 and 2002. In addition, over that same period, track widths are projected to decrease from 2 μm±0.2 μm to 400 nm±40 nm and pole-tip recessions from 5 nm±0.5 nm to 1 nm±0.1 nm [50].

##### 2.2.2.4 Microelectronics Industry

For the electronics industry, the global economy means an environment more competitive than ever, with the key to United States success seen as the development of new, breakthrough technologies [46]. The historical trends for the key product areas of dynamic random access memory (DRAM) bit count and central processing unit (CPU) performance indicate continuing reduction in geometric dimensions. In accordance with Moore’s law, minimum feature size is expected to decrease from 200 nm in 1997 to less than 100 nm after 2003. The National Technology Roadmap for Semiconductors, produced by the Semiconductor Industry Association (SIA), seeks what it calls a three-standard-deviation control of 20 nm for gate critical dimensions for the current 250 nm generation of semiconductors and 10 nm for the 130 nm generation in the year 2003 [51]. For these two levels of control, SIA specifies three-standard-deviation metrology precisions of 4 nm and 2 nm, respectively. Particularly challenging for the industry is the task of producing chips by the year 2006 with 100 nm features using non-optical lithography. Measurement is viewed as one of the five most difficult challenges it is facing [51].

### 2.3 Current Work

To address industry requirements such as those indicated above, NIST is carrying out a program of research and services at scales of dimensions from the macroscopic to the atomic.

#### 2.3.1 Large-Scale Coordinate Metrology

The focus of this work is to develop methods and capabilities to support industries—including the aircraft, ship-building, construction-and-farm equipment, and automotive—that need to make measurements of sub-meter to multiple-meter parts and structures with low, well-characterized measurement uncertainties [52]. The creation and rise in the use of discrete-point coordinate measuring systems (CMS) poses an immense problem in ascertaining the uncertainty of measurement results associated with such systems. Part of the problem is due to the large sets of numerical coordinate positions that a CMS can produce as output compared to the simpler go/no-go indications of traditional gaging. Another part of the problem is the absence of standardized methods for the characterization of the measurement performance of a CMS. One aspect of NIST’s approach to the problem is to develop computational models of the measurement uncertainty of CMSs, beginning with the older and more widely used type, the coordinate measuring machine (CMM). This work is addressing a selected set of CMMs, operated in favorable environments, measuring idealized parts. The other aspect of NIST’s approach is to develop techniques for the characterization of the measurement performance of the newer, frameless type of CMS, such as theodolite and laser-tracker systems. Related to this work is research on absolute-distance interferometry using scanned-wavelength diodes, which allow point-and-measure determinations of distance without the requirement for uninterrupted beams as in single-wavelength displacement interferometry [53].

#### 2.3.2 Dilatometry

The focus of this work is to develop a laboratory capability to measure the coefficient of thermal expansion (CTE) of materials of gages and prototype parts [52]. The goal is to support industry calibration and use of gages and artifacts at temperatures other than standard temperature. By international agreement, 20 °C is the temperature, and the only temperature, at which length dimensions of manufactured parts are defined [54]. For a measurement made at a non-standard temperature, the length at 20 °C must be calculated using the coefficients of thermal expansion of the particular gage and parts. In many cases, the uncertainties of factory measurements are limited by the uncertainty in the coefficient of thermal expansion of either the master gages or the part itself. Currently, there is no commercial or government calibration of CTEs of precision gages or parts available in the United States. NIST’s approach is to (1) develop a dilatometer to allow the measurement of the CTE of virtually any material; and (2) explore the variability of the CTE in classes of materials, including different gage materials.

#### 2.3.3 Complex Form Metrology

The focus of this work is to develop the capability to make low-uncertainty measurements of industrially important artifacts having regular geometrical forms other than the simple geometries of planes (gage blocks), cylinders (gage wires) and spheres (gage balls) [52]. NIST’s approach is to apply the technique of substitute-geometry decomposition. In this approach, the complex geometry of a part is represented as being composed of the sum of simpler geometric elements; for example, an involute as being composed of a circle of a specific radius and an offset of a specific distance. Comparator measurements then made between master artifacts of the simple forms and the elements of the more complex part. Applied successfully to the less complex forms of ball-bar artifact standards and prototype helical gears, the technique is to be extended to the more complex forms of helical gears and threads.

#### 2.3.4 Microform Metrology

The focus of this work is to develop the means to measure complex, 3D surface features at the micrometer scale that need to be quantified for their shape and size with measurement uncertainties compatible with tolerance requirements [52,55]. One of the requirements for microform metrology comes from U.S. and international work in Rockwell hardness standardization. Rockwell C hardness (HRC) is the most widely tested materials property for metal products. NIST’s approach is to develop a microform calibration system using a stylus to measure dimensions, angles, profile deviations, and alignment errors, as well as surface roughness. The work is aimed at verifying the geometric correctness of the Rockwell indenters as an alternative to hardness performance comparisons. NIST standard indenters, combined with the use of the NIST standard testing machine and a standardized testing cycle, are being used to create, maintain, and reproduce the metrology-based Rockwell hardness scale in the United States and overcome any errors that might exist in the European Community’s performance-based HRC scale [56].

#### 2.3.5 Surface Finish Metrology

The focus of this work is to develop the capability to perform state-of-the-art measurements of the microtopography of surfaces, commonly referred to as the surface finish, a dimensional feature important to the function of a wide range of industrial products [52,57]. NIST’s approach is to develop instrumentation, artifacts, and theoretical-statistical algorithms for the characterization of surface finishes using stylus profiling instruments, phase-measuring interference microscopes, and scanned probe microscopes. Issues being addressed include improved understanding of the differences between surface finish measurements performed using different types of instruments and the measurement of step-height calibrations using independently traceable techniques.

#### 2.3.6 Two-Dimensional Metrology

The focus of this work is to develop measurement algorithms, data analyses techniques, and sensor metrology for micro- and nano-meter-uncertainty calibration and use of two-dimensional positional grids to support the microelectronics and related industries [52,42]. In the United States, the need for low uncertainty artifacts to test the machines is met partially by one-dimensional calibrations of line scales or single lines of grid plates. NIST’s approach includes

- development of the measurement algorithm, method of data analysis, and sensor metrology needed to locate the grid position
- organization of an industry working group that can work towards a consensus industry standard for characterizing the measuring machines
- use by industry of a standard grid pattern, common measurement and data analysis procedures, 2D measurements on industry instruments, and NIST 1D measurements

Prototypes of this standard grid have been made and circulated to industry laboratories to obtain a baseline estimate of the current industry capabilities. The eventual goal is to have a Standard Reference Material (SRM) gridplate that will be measured by industry under NIST direction, checked with a NIST measurement of some subset of grid points, and made available to industry.

#### 2.3.7 Optical Metrology

The focus of this work is to develop the capability to perform state-of-the-art, optical-microscope-based dimensional measurements to address the measurement needs of industries that use optical-microscopes for measurement of microelectronic and related devices [52,58]. The measurements being made by industry include pitch (distance between similar-facing edges of successive graduations), linewidth (distance between opposite-facing edges of a single feature), and overlay (a hybrid feature associated with the mis-registration of successive planar levels on a microelectronic device). The NIST approach to advancing this field includes development of instrumentation, artifacts, and theoretical models of probe-artifact interactions affecting the uncertainty of measurements for confocal, reflection, and transmission optical microscopes operating with visible and UV light [58].

#### 2.3.8 SEM Metrology

The focus of this work is to develop the capability to perform state-of-the-art, scanning-electron-microscope-based, dimensional measurements to address the measurement needs of industries that use SEMs for electron-beam-lithography fabrication and SEM-based measurement of microelectronic and related devices [52,59]. NIST’s approach is to develop

- dimensional-metrology scanning-electron-microscope (SEM) instrumentation to allow low-uncertainty measurements directly traceable to the SI unit of length
- prototype calibration artifacts of appropriate materials and geometries
- Monte-Carlo simulations of the electron-beam and SEM-artifact interactions in measurements of line-widths as critical dimensions in microelectronic devices [59].

#### 2.3.9 Scanned Probe Microscope Metrology

The focus of this work is to develop the capability to perform state-of-the-art, scanned probe microscope (SPM)-based, dimensional measurements to address the measurement needs of industries that use SPMs for fabrication and metrology in manufacturing and R&D [52]. SPMs include scanning tunneling microscopes (STMs) and atomic force microscopes (AFMs) with the former operating by means of quantum-mechanical tunneling of electrons and the latter by means of interatomic forces. Both sense the distance of its probe above a surface with sensitivities at nanometer-to-picometer levels of resolution. Accurate SPM measurements are particularly important to the semiconductor, data storage, and related microfabrication industries. The most common measurements performed by such SPM users are pitch (lateral feature separation), step height (vertical surface separation), critical dimension (feature width), and surface roughness (often specified using the root-mean-square roughness parameter). A calibrated AFM (C-AFM) has been developed to extend pitch measurements to sub-micrometer pitch values and below [24]. An STM-based “Molecular Measuring Machine” (M3) has been developed to make nanometer-level measurements over a 50 mm by 50 mm area [36]. In addition, extensive work has been carried out on accounting for the effect of the finite size and geometry of the scanning tip in dimensional measurements made with SPMs [38,60].

#### 2.3.10 Atom-Based Artifact Standards

The focus of this work is to develop a family of dimensional artifact standards for which the dimensional properties of the artifact derive from atomic-scale material properties and, as a result, features have inherent nanometer- and sub-nanometer-scale dimensions and geometric perfection [52]. The relevant types of dimensional features of these atom-based artifact standards include counted-atom linewidths and lattice step heights. NIST’s approach is to

- use atomic-scale material deposition processes and controlled surface modification to fabricate artifacts that have features with highly-controlled atomic-scale dimensions based on the structure of the crystal lattice
- measure and statistically verify geometry and dimensions using metrology atomic-force and scanning-tunneling microscopes tied directly to the SI unit of length.

Target feature dimensions and uncertainties for these future atom-based standards are:

- linewidths of 300 nm and expanded uncertainty (coverage factor *k* = 2) of 3 nm
- step heights of 300 pm and expanded uncertainty (coverage factor *k* = 2) of 10 pm.

Work in this area includes development of methods to determine linewidth based on counting of atom spacings across a line [61] and algorithms for calculating single-atom step heights [62].

#### 2.3.11 Atomic-Scale Displacement Metrology

The focus of this work is to develop the laboratory capability to precisely generate and accurately measure displacements in increments of 50 pm over distances of tens of centimeters. The intended result is improvement by an order of magnitude upon the approximate relative uncertainty of 5×10^−8^ that forms the practical lower limit in long length measurements done using displacement interferometry in air. Such capability is to be based in part on advancing the state-of-the-art of displacement interferometry by a direct intercomparison of x-ray, Fabry-Perot, and optical-heterodyne interferometry. In addition, it is to be based on long-range high-precision stages. These stages are to incorporate laser-based metrology; control of translation, pitch, and yaw, and positional capability commensurate with pm-level displacements. The resulting system of interferometry and stage will form the prototype for production and metrology stages of the future and is ultimately intended to be the basis of a next-generation linescale interferometer system for the measurement of linescales of lengths from less than 1 μm to 1 m [63].

## 3. The Future

The future of length and dimensional metrology is being shaped by theoretical and practical limits to attainable uncertainties in measurement, by continuing trends in industry, and by the emerging response of NIST as an institution to those limits and trends.

### 3.1 Limits: Ultimate, Standards-Based, and Practical

There are two sources of pressure for the achievement of ever-smaller uncertainties in length and dimensional measurements. These are, first, the continuing industrial trend to tighter tolerances—represented in the microelectronics domain by Moore’s Law—and, second, the continuing scientific trend to explore the limits of understanding through physical measurement. Given these drivers, a question that arises is whether there are theoretical and practical limits to the lowest uncertainty that may be achieved. The following sections discuss such lower limits—ultimate-theoretical, standards-based, and practical.

#### 3.1.1 Ultimate Theoretical Limits

With the continuing evolution of technology, fundamental physics may impose limits on the uncertainty of measurements of length. At the forefront of today’s experimental research in cosmology, quantum physics, relativity, and fundamental particles the question of ultimate theoretical limits is an immediate one.

##### 3.1.1.1 Quantization of Space

The space of virtually all of current applied physics, engineering, and, hence, commerce is the space of Newtonian and relativistic mechanics. At this macroscopic level, space is a homogeneous continuum and no structure of space poses a lower limit to uncertainty of measurements of length. At the microscopic level, however, such may not be the case. Quantum effects become important and both gravity and the structure of space itself may be quantized. Much work is underway in the science community to explore these possibilities, which are expected to occur at dimensions of the order of the Planck length, 10^−35^ m [64].

##### 3.1.1.2 Heisenberg Uncertainty Principle

The Heisenberg uncertainty principle (HUP) does not place an ultimate limit on the uncertainty of measurement of position per se. However, it does set an ultimate limit on the simultaneous, and successive, measurements of special pairs of measurement quantities, one of which includes position [65]. According to the HUP, a measurement of the momentum of an object must disturb its position and a measurement of its position must disturb its momentum. The result is that the more accurately that momentum is known, the less accurately can its position be known. The HUP limit is given by

Δx×Δp≥ℏ/2, (1)

where Δ*x* is the uncertainty in position, Δ*p* is the uncertainty in momentum, and ℏ is the Planck constant divided by 2π. The effect of the HUP limit was encountered in efforts to detect cosmic gravitational waves. In that experiment, measurements of the change in position as small as 10^−21^ m of detectors weighing up to 10 metric tons needed to be made at time intervals of τ = 10^−3^ s. For these conditions, the HUP set a limit in the uncertainty in successive measurements of position of Δ*x* approximately 5×10^−21^ m, five times worse than that desired [66].

##### 3.1.1.3 Johnson *kT* Noise

There is a dimensional equivalent of Johnson, or thermal, noise that places an ultimate limit on the uncertainty of measurement of dimensional features [67]. Johnson noise in an electronic circuit is the variation in the voltage across a conductor due to thermal agitation of the electrons passing through it [68]. This Johnson noise is proportional to (*RkT*)^1/2^ where *R* is the resistance, *k* is the Boltzmann constant and *T* is the thermodynamic temperature. Thermal length fluctuations of a solid, the spatial equivalent of electronic Johnson noise, are due to thermal agitation of the atoms of the material. In a measuring machine, such thermal noise places an ultimate limit on the location of the origin of the axes of the machine and, therefore, on the uncertainty of position measurements the machine can attain. Thermal noise similarly limits the uncertainty with which the length of an object can be measured. For example, for a homogenous isotropic cube, the root-mean-square (rms) thermal fluctuation Δ*l* in the length *l* of the side of the cube is given by

$$\Delta l={\left(kT/3B\;l\right)}^{1/2},$$ (2)

where *B* is the bulk modulus of the material of the cube. Note that this contribution to the uncertainty in the measurement of the length of a material object is inversely proportional to the length and thus becomes more and more important at smaller and smaller scales. For example, for an object with a bulk modulus of that of fused silica, 3.5×10^10^ N/m^2^, and a temperature of 300 K, the rms fluctuation in dimension of a 1 m cube is 0.2 fm (10^−15^ m) or, fractionally, 2×10^−16^. The rms fluctuation in a 1 nm cube is 6.3 pm (10^−12^ m), fractionally 6×10^−3^ or 0.6 % [67].

#### 3.1.2 Limits from Primary Reference Standards

Two other reference standards for SI units place ultimate limits on the uncertainty with which measurements of length can be made. These are the reference standards for the practical realization of the second as the unit of time and for the kelvin as the unit of temperature.

##### 3.1.2.1 Primary Reference Standard for the Second and for the Meter

While an independent unit, the meter, the SI base unit of length, is now defined in terms of the speed of light in vacuum and an interval of time. As a result, a limit for uncertainty of measurements of length in principle is set by the uncertainty with which the second, the SI base unit of time, can be realized. The primary standard for interval of time is an array of atomic clocks located at national metrology institutes and the International Bureau of Weights and Measures (BIPM). The current relative standard uncertainty associated with the timescale based on this array of atomic clocks is 1.5 to 5×10^−15^ [69]. The uncertainty of the NIST cesium primary frequency standard is estimated to be 1.8×10^−15^ [70]. However, the de facto primary-standard limit for practical SI-unit measurements of length is not the cesium atomic clock itself, but another frequency standard referenced to that clock, the iodine-stabilized helium-neon laser. The current relative standard uncertainty for the 632.99 nm line of an iodine-stabilized helium-neon laser, the work-horse reference standard for practical metrology that conforms to the CIPM prescription for design and operation, is 2.5×10^−11^ [4].

##### 3.1.2.2 Temperature Standards and Length of Material Objects

Materials expand and contract with changes in temperature. However, by international agreement, the reference temperature at which the length of a material object is defined is 20 °C. As a result, the uncertainty with which the International Temperature Scale for 1990, ITS-90, can be implemented at 20 °C sets another primary-standard limit for uncertainty of measurements of material length. The current reproducibility of ITS-90 at the length-standard reference temperature of 20 °C is 0.0001 °C. Given that the change in length Δ*L* of a material object of length *L* with coefficient of thermal expansion *α* at *t* = 20 °C for a change in temperature Δ*t* is given by:

ΔL/L=α⋅Δt, (3)

then the current thermal limit for determination of the length of a material at the reference temperature of 20 °C with a coefficient of thermal expansion in the range from 2.5 to 25×10^−6^/°C, corresponding approximately to silicon and aluminum, is fractionally 2.5×10^−10^ and 2.5×10^−9^, respectively. As such, in terms of relative expanded uncertainty (coverage factor *k* = 2), the current temperature-defined limit for the determination of the length of a body of common materials is of the order of 5×10^−10^ [71].

#### 3.1.3 Practical Limits

Away from the strictly controlled laboratory conditions of national metrology institutes, where measurements of spatial quantities are made at world state-of-the-art capability, measurement uncertainties are more often determined by practical, rather than ultimate, limits.

##### 3.1.3.1 Displacement Interferometry

Optical-wavelength displacement interferometry, which forms the practical basis for SI-based measurements of length, is limited in practice by variations in the index of refraction of the medium, typically air, through which the laser light beam propagates in the course of the measurement. Since the index of refraction of a gas is a function of its temperature, pressure, humidity, and chemical composition, the uncertainty for optical interferometric displacement measurements made without compensation for actual variations in those parameters can be large. For example, a fractional length error of 1×10^−6^ would result from any one of the following variations: a 1 °C change in temperature, a 0.33 kPa (2.5 mm Hg) change in atmospheric pressure, or an 80 % change in relative humidity [72]. Using the Edlen formula (an internationally agreed upon equation for the calculation of the index of refraction of typical laboratory air as a function of wavelength, air temperature, air pressure, and relative humidity), compensation can be made for these variations. As a result, a practical lower limit for the fractional uncertainty of laser displacement measurements is estimated to be 1.2×10^−7^. The fractional uncertainty in the Edlen formula itself is estimated to be 5×10^−8^, which forms the practical lower limit of long length measurements done using displacement interferometry in air [72]. For short lengths, the practical combined-standard-uncertainty limit of optical heterodyne interferometry, whether in air or vacuum, due to all factors (including internal reflections, mixing of polarization states, and diffraction), has been estimated to be 0.1 nm [67].

##### 3.1.3.2 Probe Limitations

Probing as the means to detect the boundary of an object places practical limits on uncertainty attainable in dimensional measurements. One source of this uncertainty is uncompensated variations in the effective location of the probe as it interacts with the object boundary. For a dimensional measurement, probing of two successive boundaries is required. For a measurement of displacement, distance, or position, some of the systematic errors of probing of the successive boundaries are subtractive, cancel out, and do not contribute to the measurement uncertainty. For a measurement of an extension, some of these same systematic errors are additiv and increase the overall error or uncertainty of measurement. Table 3 shows representative uncertainties in measurements of feature spacings and widths due to probe-object interactions with progressively higher resolution probes, including mechanical-contact coordinate measuring machines [34] and optical, scanning electron, and scanning tunneling microscopes [67].

##### 3.1.3.3 Temperature

The dependence of the length of a material body on temperature is such an important effect in industrial length metrology that temperature uncertainty is very often the practical limiter of uncertainty.

Table 4 shows length measurement uncertainties associated with the limiting uncertainties attainable with state-of-the-art temperature measurement by different types of thermometry for a material with an assumed coefficient of thermal expansion of 10×10^−6^/°C, which corresponds approximately to that of steel. These types of thermometry are [71]

- a standard platinum resistance thermometer (SPRT) immersed in a gallium melting-point cell
- and SPRT as a sensor referenced to another primary-calibrated SPRT via a resistance bridge
- a thermocouple (TC) referenced to an SPRT by a bridge
- a thermistor
- a mercury(Hg)-in-glass thermometer
- a thermocouple.

Table 5 shows the standard uncertainties and relative standard uncertainties, *u*(*L*) and *u*_r_ = *u*(*L*)/*L*, for representative laboratory and industrial measurements of length *L* with different degrees of temperature control and nearness to standard temperature. The uncertainties correspond to realistic limiting conditions of measurement in industrial and standards-laboratory applications [71]. For such measurements, there are two contributions to the overall uncertainty *u*(*L*) in a measured material length. First, there is the contribution due to the variation in temperature, which is proportional to the coefficient of thermal expansion *α* and the uncertainty in the temperature *u*(*t*). Second, there is the contribution due to the uncertainty in the value of the thermal expansion, which is proportional to the uncertainty in the coefficient of thermal expansion u(*α*) and the difference, *t−*20 °C, between the actual temperature *t* and the standard temperature *t*_0_. When combined in quadrature, the overall combined standard uncertainty in length measurements made at a non-standard temperature is given by:

$$u\left(L\right)=L\cdot {\left[{\left\{\alpha \cdot u\left(t\right)\right\}}^{2}+{\left\{u\left(\alpha \right)\cdot \left(t-20{}^{\circ}C\right)\right\}}^{2}\right]}^{1/2}$$ (4)

The second and third columns show representative practical limits of length measurement uncertainties due to temperature associated, for example, with the manufacture of a high-quality, aluminum automobile-engine piston and a steel precision lead screw under the conditions specified. Corresponding uncertainties in distance measurements under conditions representative of tertiary, secondary, and primary length-standards laboratories are shown in columns three, four and five. The last column in the table shows aspects of a nm-uncertainty measuring machine currently under development [36]. Achievement of a combined standard uncertainty of 1 nm in measurement of a distance of 70 mm on a silicon substrate with no more than 0.1 nm contributed by thermal effects requires a temperature uncertainty and an average temperature difference from standard temperature of less than of 0.001 °C [36].

### 3.2 Industry Trends and Emerging NIST Responses

According to one set of manufacturing industry watchers [46], the highest-level macroscopic trends expected to dominate the opening of the 21st century are

- the globalization of markets and business competition
- the accelerating pace of change in technology
- the rapidly expanding access to technology
- the ubiquitous availability and distribution of information
- the increase of customer expectations

Coupled to these highest-level trends are two strong intermediate-level trends expected to affect the areas of concern of this article: a further Moore’s-Law-like tightening of tolerances on manufactured products and a shift to greater emphasis on international industry standards (over national industry standards). Three lower-level trends are specifically expected to shape the research, measurement services, and standards-commit tee activities of NIST in the area of length and dimensional measurements in the immediate future. These are emergence of new technologies, increased demand for calibration artifacts, and development of the ISO Global Product Specification chain of standards [52].

#### 3.2.1 Emergence of New Traceability

Historically in the United States, traceability—the “old traceability”—was driven virtually exclusively by defense procurement and regulatory safety requirements and could be satisfied in a pro forma manner. Currently, traceability—the “new traceability”—is being driven by commercial markets and is being specified in international product standards aimed to be more than pro forma in nature. This new traceability is a requirement that a buyer of dimensioned parts imposes on the manufacturer of those parts, either directly through a part specification or indirectly through a quality-management specification. The condition is that measurements on manufactured parts made to show conformity of part dimensions to buyer’s specifications must be traceable. To exhibit the new traceability, measurement must be referenced to the international standard of length through a well-documented and unbroken chain of timely [73] task-specific [74] comparisons. Both the results of measurements and the uncertainties of the measurement results need to be shown for each comparison in the chain [27].

#### 3.2.2 Increasing Demand for Calibrated Artifacts

Coupled to the accelerating rate of change of technology and to the new traceability requirements in international standards is U.S. industry need for new and improved, physical-artifact, dimensional standards applicable to industry-specific requirements. Two motivators for lower-uncertainty artifact standards are commonly cited by U.S. manufacturing companies. First, there is a need for traceability to meet ISO-9000-type quality requirements for products to be sold both in the European Economic Community and in the Pacific-rim nations. Second, there is a need for low-uncertainty references to support development of innovative, high-technology products comparable to those developed by Japan [75].

#### 3.2.3 Development of GPS Chain of Standards

Coupled with the trend toward globalization of markets and the rise of international over national industry standards is development of an all-encompassing set of ISO Standards on “Geometric Product Specifications (GPS)” [74]. This family of standards deals in detail with verifying that measurements made on a manufactured part insure conformity of the part to design specification. The GPS standards cover all of dimensional features indicated on a technical drawing, such as size, distance, position and surface roughness, and all related measuring instruments and their calibration. It is the GPS that requires that results of dimensional measurements made on a manufactured part have an associated uncertainty specific to the type of part feature measured in a particular way (“task-specific”) and be traceable to the international standard of length [76].

### 3.3 The Evolving NIST Response

To meet anticipated needs of U.S. manufacturing industries, NIST is undertaking alternatives to the traditional NMI as the top of a classical hierarchy of calibrations.

#### 3.3.1 Atom-Based Artifact Standards

**Fig. 3:**
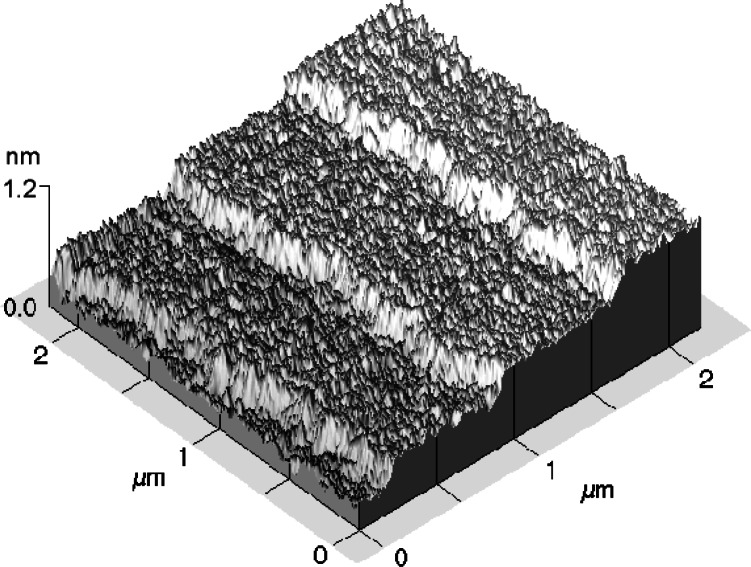
NIST calibrated atomic force microscope (C-AFM) image of single-atom step heights on a silicon (111) lattice [24].

As an alternative to traditional artifact standards, NIST is seeking to do for nano-scale dimensional metrology what the redefinition of the meter did for length, allow a move from man-made prototype standards to constants of nature as intrinsic standards. Historically, precision artifact standards—from gage blocks to photomask linewidths—have received their form by material-shaping manufacturing processes and received their dimensional values by an independent calibration. The objective of this future-oriented work is to achieve a family of dimensional standards that receive their form and dimensions from the atomic lattice [44,45]. Figure 3 shows the first prototype of such a standard, one in which the atom-spacing of the Si (111) lattice provides the form and dimension of a step height standard [24].

#### 3.3.2 Use of Other Government Capabilities

In one case, an alternative to NIST’s use of its own equipment and staff as the sole basis for NIST dimensional-measurement calibration services, NIST is using those of another Federal agency. NIST now provides calibrations of industrial step gages using equipment and staff of the Department of Energy’s government-owned, contractor-operated Y12 facility in Oak Ridge, Tennessee, under the administrative and metrological control of NIST. In addition, NIST is metrologically supporting that facility’s own provision of NIST-traceable calibrations in gear form and other dimensional quantities. It does so through its collaboration in the joint NIST-DoE/Y12 Metrology Center at Y12. This overall endeavor has been honored with two U.S. Vice President’s Hammer Awards for overcoming institutional barriers to excellence in provision of customer service, one in each of the two areas of collaboration [77].

#### 3.3.3 Use of Industry Capabilities

As one alternative to development of a NIST Standard Reference Material and acquisition of multimillion-dollar measuring machines, NIST has initiated industry efforts to achieve NIST-traceable calibrations of next-generation 2D grids for the microelectronics industry without NIST calibration of 2D grids. It has done so by a combination of actions [42]. First, it led the formation of a standards committee to define a standardized pattern for a 2D grid standard. Then it organized an industry working group to agree upon the design and procedures for measurement and data analysis procedures for a 2D grid standard. It arranged use of industry equipment for measurements of the 2D grid. And, finally, it performed 1D measurements to provide the link to the SI unit of length.

#### 3.3.4 Shop Floor as NMI

Finally, NIST is pursuing an alternative to its development of task-specific measurement services, capabilities, and methods. NIST has initiated a program of research and standards-committee activities to support industry’s ability to carry out task-specific dimensional measurements without recourse to NIST calibrated dimensional standards. For such to be the case, industry needs standardized means to carry out dimensional measurements on the manufacturing shop floor that

- are directly and immediately traceable to the SI unit of length
- have uncertainty statements that comply with the ISO “Guide to the Expression of Uncertainty in Measurement”
- are able to satisfy the requirements of the emerging ISO GPS chain of standards
- are able to satisfy the quality system requirements of ISO 9000, ISO 17025, and NCSL Z540-1

all without recourse to NMI-calibrated dimensional standards [78]. NIST’s approach is to carry out R&D on non-task-specific measurement techniques and support development by industry of documentary standards, which taken together would allow industry to meet the conditions above without the need for NIST task-specific reference standards.

## 4. Conclusion

This paper has discussed the past, present, and future of length and dimensional measurements at NIST. It has examined the evolution of the SI unit of length through its three definitions, including the contributions of NIST to the redefinitions through work on mercury-198 pressure lamps and iodine-stabilized helium-neon lasers as reference wavelength standards. It has also examined the evolution of dimensional metrology since 1901, including the contributions of NIST in that field. NIST’s historical achievements include its work on precision gage blocks, software-error correction of coordinate measuring machines, optical and SEM photomask linewidth standards, and the first scanned-probe microscope (the basis for the Nobel-Prize-winning scanning tunneling microscope). Current work the paper describes includes a broad range of measurement technologies from 100-meter-range laser-trackers to picometer-resolution displacement interferometers. Finally, it has looked at trends for the future. These trends suggest that the first decade of the second century for NIST may be governed by a search for alternative ways to meet the challenging technological needs of the United States for NIST measurement services.

## Figures and Tables

**Table 1:** Ranges and uncertainties of selected NIST dimensional measurement capabilities

Measurement types	Range (*L*_min_ to *L*_max_)	Uncertainty *U* (*k* = 2)	*U*/*L*_min_	*U*/*L*_max_	Relative to leading NMI
Linescales					

Measuring tapes [20]	1 m to 50 m	60 μm to 500 μm	6 × 10^−5^	1 × 10^−5^	Tied with leader
Linescales (“long”) [11]	10 μm to 1 m	1 nm to 70 nm	1 × 10^−3^	7 × 10^−8^	Leader
Linescales (“short”) [11]	1 μm to10 μm	1 nm	1 × 10^−3^	1 × 10^−4^	Leader

End standards					

CMM step gages [21]	100 mm to 1 m	0.4 μm to 0.7 μm	4 × 10^−6^	7 × 10^−7^	Tied with leader
Gage blocks [22]	1 mm to 100 mm	10 nm to 30 nm	1 × 10^−5^	3 × 10^−7^	Same as leading NMIs
IC photomask linewidth [23]	0.5 μm to 30 μm	36 nm	7 × 10^−2^	1.2×10^−3^	Leader
Step height [24,25]	300 pm to 75 μm	8 pm to 0.4 μm	2.5 × 10^−2^	5 × 10^−3^	Leader

**Table 2:** NIST dimensional measuring machines for first-principles measurements of dimensions

Measuring machine	Probe	Frame	Scales	Wavelength reference
CMM^a^	Mechanical contact	*x-y* stage *z*-ram	*x*, *y* & *z* interferometers	HeNe
Gage-block interferometer	Visible light	Platen, bridge	*z* (Michelson) interferometer	HeNe
Overlay microscope	Visible light	*x*-*y* stage *z*-PZT	*x*, *y* & *z* interferometers	HeNe
Metrology SEM	Electron beam	*x*-*y* stage	*x* interferometer	HeNe
Calibrated AFM	Atomic force	*x*-*y* stage *z*-PZT	*x* & *y* interferometers *z* interferometer-calibrated CG	HeNe
M3	Scanning tunneling	*x*-*y* stage *z*-PZT	*x* & *y* interferometers *z* interferometer-calibrated PZT	HeNe

a CMM: coordinate measuring machine; HeNe: helium-neon laser; PZT: piezo-electric transducer; SEM: scanning electron microscope; AFM: atomic force microscope; CG: capacitance gauge; M3: Molecular Measuring Machine.

**Table 3:** Representative combined standard uncertainties in measurements of feature spacings and widths due to probe-object interactions (coverage factor *k* = 2 assumed) [34,67]

Type of probe	Probe-object interaction	Uncertainty in feature spacing	Uncertainty in feature width
Mechanical-contact CMM^a^	Mechanical deformation	0.2 μm	0.5 μm
Optical microscope (OM)	Optical diffraction	0.045 μm	0.065 μm to 0.65 μm
Scanning electron microscope (SEM)	Electron scattering	4 nm	6 nm to 60 nm
Scanning tunneling microscope (STM)	Quantum vacuum tunneling	0.014 nm	0.15 nm to 0.2 nm

a Coordinate measuring machine.

**Table 4:** Lenght measurement uncertainties associated with the limiting standard uncertainties of temperature measurement by different forms of thermometry (coverage factor *k* = 1)

Sensor element^a^	Reference element	Reference instrument	Temperature uncertainty	Length uncertainty at 1 m for steel
SPRT	Ga-Pt		0.0001 °C	1 nm
SPRT	SPRT	Bridge	0.001 °C	10 nm
TC	SPRT	Bridge	0.002 °C	20 nm
Thermistor		Bridge	0.01 °C	0.1 μm
Hg			0.03 °C	0.3 μm
TC		DVM	0.1 °C	1 μm

a SPRT: Standard platinum resistance thermometer; TC: thermocouple; Hg: mercury-in-glass thermometer; DVM: digital volt meter.

**Table 5:** Length measurement uncertainties associated with different degrees of temperature measurement and control attainable in principle at temperatures near but not exactly at the standard temperature *t*_0_ of 20 °C

	Engine piston	Lead screw	Tertiary laboratory	Secondary laboratory	Primary laboratory	R&D device
*L*	100 mm	1000 mm	1000 mm	1000 m	1000 mm	70 mm
Material	Aluminum	Steel	Steel	Steel	Steel	Si
*α* (10^−6^/°C)	23.4	11.8	11.8	11.8	11.8	2.6
*u*(*t*)	10 °C	1 °C	0.1 °C	0.01 °C	0.001 °C	0.001 °C
*u* (*α*) (10^−6^/°C)	0.7	0.7	0.035	0.035	0.035	
*t–t*_0_	3 °C	3 °C	1 °C	0.1 °C	0.01 °C	0.000 °C
*u*(*L*)/*L*	2.3×10^−4^	1.2×10^−5^	1.2×10^−6^	1.2×10^−7^	1.2×10^−8^	2.6×10^−9^
*u*(*L*)	23 μm	12 μm	1.2 μm	0.12 μm	12 nm	0.2 nm
